# Tip-in underwater endoscopic mucosal resection for a sessile lesion at a poorly visualized location

**DOI:** 10.1055/a-2764-4824

**Published:** 2026-01-20

**Authors:** Keijiro Numa, Kenichiro Imai, Kinichi Hotta, Kazunori Takada, Sayo Ito, Hiroyuki Ono

**Affiliations:** 1 38471 Division of Endoscopy, Shizuoka Cancer Center, Shizuoka, Japan

**Fig. 1:**
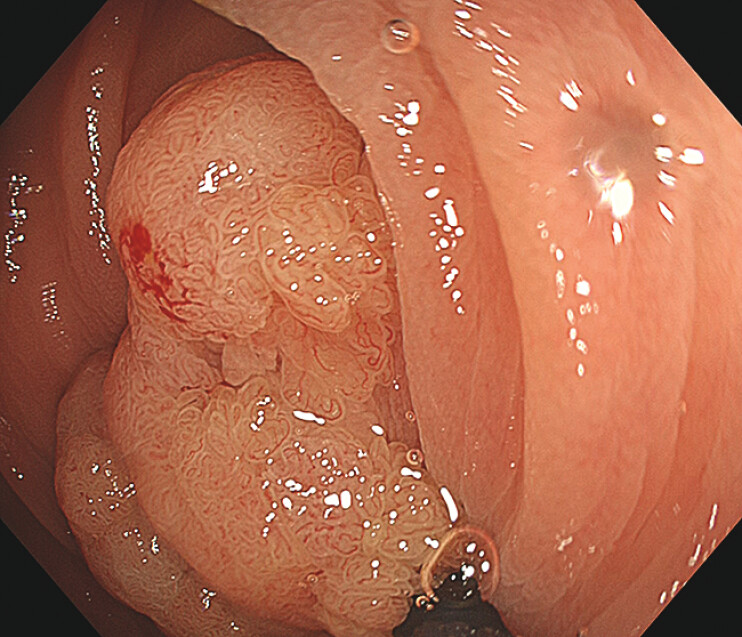
An endoscopic image of a 19-mm sessile lesion at the hepatic flexure.

Endoscopic resection of proximal colonic lesions located behind the flexure is challenging because poor visualization of the oral side of the lesion makes it difficult to capture the entire lesion. Water immersion improves scope maneuverability and alleviates luminal angulation by reducing luminal distension 1 . Thus, underwater endoscopic mucosal resection (UEMR) could be effective for poorly visualized polyps such as orifice- or diverticular-related polyps 2 3 . Nevertheless, suboptimal visualization of proximal margins in the deflated lumen would hamper assured snaring in UEMR 4 . We present a case of successful en bloc resection using tip-in UEMR of a proximal colonic lesion with poor visualization due to the location behind the flexure ( Fig. 1 ).

**Fig. 2:**
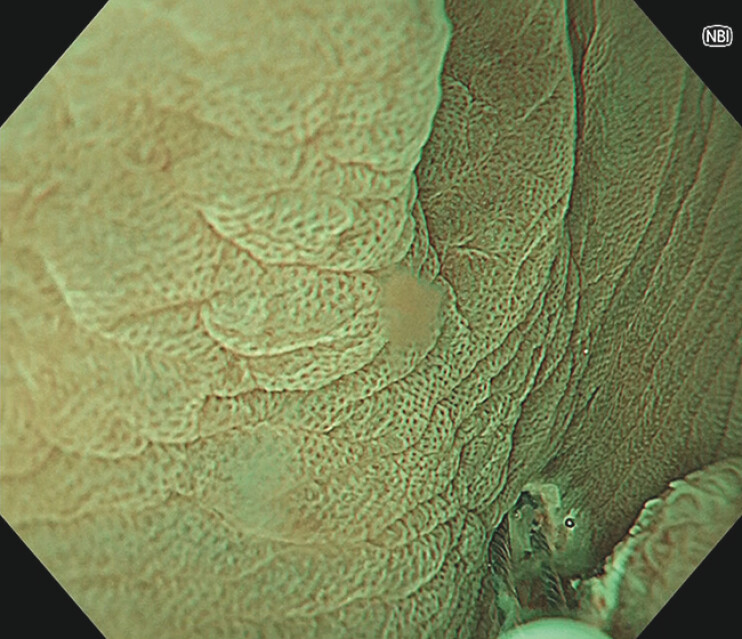
The snare tip was anchored at the proximal mucosa using the cutting current to secure the resection margin and stabilize snare manipulation.

**Fig. 3:**
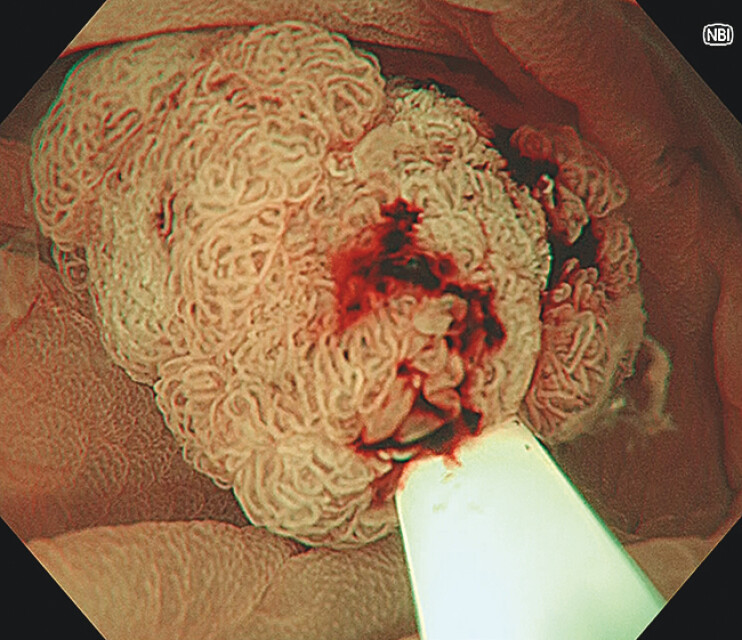
The lesion was completely captured within the snare.

**Fig. 4:**
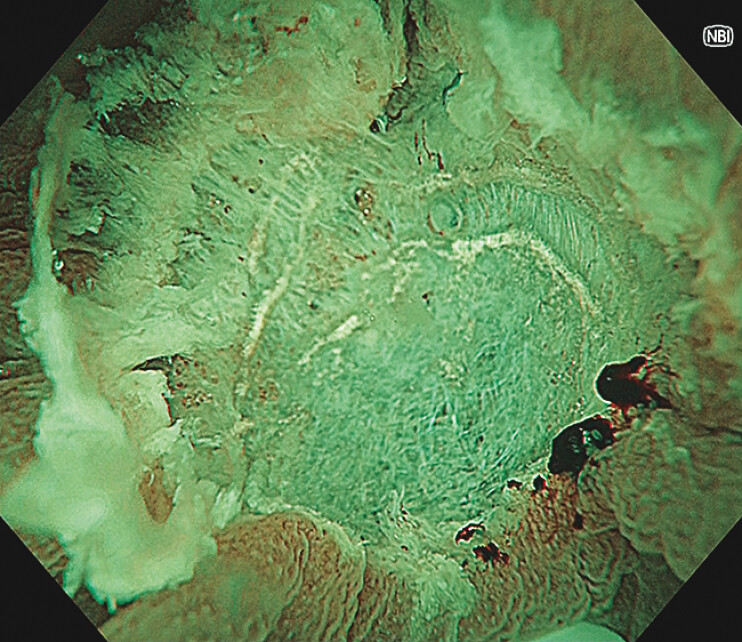
An endoscopic image showing the post-tip-in UEMR defect without residue and perforation.

**Fig. 5:**
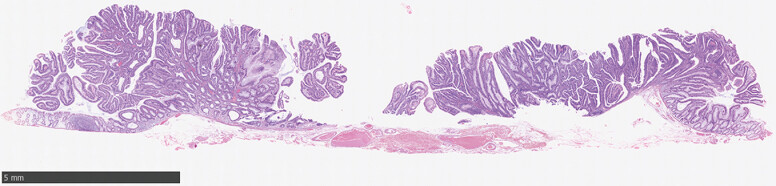
Histopathological examination showing high-grade dysplasia with negative margins.

A 68-year-old man was referred with a protruded lesion of 19 mm in size at the hepatic flexure. As the lesion was diagnosed as an adenoma, UEMR was attempted to improve scope maneuverability and its visualization. However, poor visualization of the oral side of the lesion did not ensure snare capturing of the entire lesion. To assure proximal margin, a spot-shaped mucosal incision at the proximal site of the lesion was made with a snare tip using a cut current ( Fig. 2 ), and the lesion was within the snare ( Fig. 3 ). Stable snare manipulation owing to an anchored tip could achieve en bloc resection without adverse events ( Fig. 4 , Video 1 ). Histopathological examination revealed high-grade dysplasia ( Fig. 5 ).

Tip-in underwater endoscopic mucosal resection enabled en bloc resection of a hepatic flexure lesion with poor access by applying water immersion and snare anchoring to secure the resection margin.Video 1

In this case, tip-in UEMR has several advantages. First, reduced luminal distension could improve scope maneuverability and flexure angulation. Second, the tip-in maneuver could assure proximal margins even in poor visualization. Third, the anchoring snare tip could secure the snare capturing even where scope maneuverability was poor. This case suggests that tip-in UEMR is an effective technique for polyps with poor visualization and scope maneuverability.

Endoscopy_UCTN_Code_TTT_1AQ_2AD_3AC
